# Ensuring leadership at the operational level of a health system in protracted crisis context: a cross-sectional qualitative study covering 8 health districts in Eastern Democratic Republic of Congo

**DOI:** 10.1186/s12913-023-10336-7

**Published:** 2023-12-06

**Authors:** Rosine N. Bigirinama, Samuel L. Makali, Mamothena C. Mothupi, Christian Z. Chiribagula, Patricia St Louis, Pacifique L. Mwene-Batu, Ghislain B. Bisimwa, Albert T. Mwembo, Denis G. Porignon

**Affiliations:** 1 Ecole Régionale de Santé Publique, Université Catholique de Bukavu, Bukavu, Democratic Republic of Congo; 2 School of Medicine, Université Catholique de Bukavu, Avenue Michombero No. 02, Bukavu, Democratic Republic of Congo; 3 Ecole de Santé Publique, University of Lubumbashi, Lubumbashi, Democratic Republic of Congo; 4 Centre de Recherche Politiques, Systèmes de Santé, Santé Internationale (CR3), Ecole de Santé Publique, Université Libre de Bruxelles, Bruxelles, Belgique; 5 School of Public Health, University of the Western Cape, Cape Town, South Africa; 6 School of Medicine, Université de Kaziba, Bukavu, Democratic Republic of Congo; 7 Centre de Recherche en Sciences Naturelles, Lwiro, Democratic Republic of Congo; 8 Département des Sciences de la Santé Publique, School of Medicine, Université de Liège, Liège, Belgium

**Keywords:** Leadership, Health system resilience, Sub-Saharan Africa, Crisis context, DRC

## Abstract

**Background:**

This study examines how leadership is provided at the operational level of a health system in a protracted crisis context. Despite advances in medical science and technology, health systems in low- and middle-income countries struggle to deliver quality care to all their citizens. The role of leadership in fostering resilience and positive transformation of a health system is established. However, there is little literature on this issue in Democratic Republic of the Congo (DRC). This study describes leadership as experienced and perceived by health managers in crisis affected health districts in Eastern DRC.

**Methods:**

A qualitative cross-sectional study was conducted in eight rural health districts (corresponding to health zones, in DRC’s health system organization), in 2021. Data were collected through in-depth interviews and non-participatory observations. Participants were key health actors in each district. The study deductively explored six themes related to leadership, using an adapted version of the Leadership Framework conceptual approach to leadership from the United Kingdom National Health Service’s Leadership Academy. From these themes, a secondary analysis extracted emerging subthemes.

**Results:**

The study has revealed deficiencies regarding management and organization of the health zones, internal collaboration within their management teams as well as collaboration between these teams and the health zone’s external partners. Communication and clinical and managerial capacities were identified as key factors to be strengthened in improving leadership within the districts. The findings have also highlighted the detrimental influence of vertical interventions from external partners and hierarchical supervisors in health zones on planning, human resource management and decision-making autonomy of district leaders, weakening their leadership.

**Conclusions:**

Despite their decentralized basic operating structure, which has withstood decades of crisis and insufficient government investment in healthcare, the districts still struggle to assert their leadership and autonomy. The authors suggest greater support for personal and professional development of the health workforce, coupled with increased government investment, to further strengthen health system capacities in these settings.

## Background

Advances in medical science and technology offer global opportunities for improved healthcare delivery and extended life [1]. The implementation of these interventions occurs at the country level through health systems. The World Health Organization (WHO) defines a health system as a coordinated network of elements and individuals providing holistic care to populations [2]. However, the alignment between policy options and the capacity of national health systems to deliver quality universal healthcare varies, especially in low- and middle-income countries. These countries consistently face challenges in achieving healthcare performance comparable to high-income countries [3, 4], particularly in sub-Saharan Africa [5]. Most of these countries are experiencing various crises and emergencies (humanitarian, political and security-related in particular), which largely explain their poor health performance [6–10]. In the recent global context, the COVID-19 pandemic has exacerbated underperformance, with 90% of countries reporting significant disruptions to health services [5, 11]. However, the pandemic underscored the critical role of strong leadership in enabling multisectoral coordination to prepare the health system for international crises, both at national and sub-national levels.

Health policy experts emphasize that strong leadership is a crucial element for health system development [1, 12], a prerequisite for health system strengthening and overall progress [13]. Leadership has been shown to play a pivotal role in resilience and positive health system change in various contexts [14–16].

Eastern Democratic Republic of Congo, facing a protracted crisis for over three decades, has witnessed a significant decline in socio-economic conditions, resulting in a weakened health system and a severe lack of healthcare access for the majority of the population [17]. Among the most affected regions by these ongoing conflicts is the South Kivu province in Eastern DRC. For several decades, the province served as a hotspot for armed groups and rebel movements, resulting in a volatile security environment. These conflicts have given rise to a significant humanitarian crisis, leading to widespread displacement, food insecurity, and limited access to essential services, with ethnic tensions and resource disputes further fueling the turmoil [17–19]. Weak state presence and governance challenges persist in some areas up to now, exacerbating the insecurity, while high levels of gender-based violence, often used as a weapon of war in the late 1990s and earl 2000s [20], have devastating and lasting consequences [21, 22]. Hence the need to promote the leadership is necessary to achieve greater resilience of the health system [23].

Frequently, academic health worker programs lack leadership modules [24, 25]. This gap exists in the DRC, where little is known about leadership development in the Congolese health system [26]. Some Ministry of Health reports mention a leadership training course, focusing on key functions (scan, focus, align, inspire), held over a decade ago by Management Science for Health (MSH) for chief medical officers and Ministry of Health executives. Unfortunately, there were no clear follow-up [27]. Information on the completion and employment status of these trained managers is currently unavailable. In this study, our primary objective is to assess the state of leadership at the health district level in South Kivu, Eastern DRC. We aim to investigate the extent to which leadership is developed and practiced within the Congolese health system, and to uncover the challenges and opportunities for leadership development in this protracted crisis context.

## Methods

### Settings and study period

This study was conducted in the South Kivu province in Eastern DRC. Data and events are considered for the year 2021.

The health system in the DRC is organized in three levels: (i) the Central level, where health policies, norms and strategies are elaborated in the Ministry of Health; (ii) the intermediate level represented by the 26 provinces which plays a role of coordinating health interventions, planning and technical support (through the Provincial Health Division – PHD) and of control, audit and inspection (through the Provincial Health Inspection (PHI); (iii) and the operational level featuring 516 Health Zones (HZ – corresponding to health entities commonly referred to as Health Districts ) where health policies and strategies are implemented [28, 29]. The HZ is managed by an autonomous Health Zone Management Team (HZMT) whose mission is to ensure the consistency of activities, including improving health coverage and quality of care, streamlining the operation of integrated health structures, management of human and financial resources, and organizing community participation. While the composition of HZMTs may vary, the core members often include:

- Health Zone Medical Chief or Chief Medical Officer: He is a senior healthcare administrator responsible for the overall management and leadership of the HZ.
- Medical Officer: He is a medical doctor responsible for clinical oversight, ensuring the delivery of quality healthcare services, and providing medical expertise within the HZ. He is in general the Medical Director of the General Referral Hospital (MD-GRH) of the HZ.
- Nursing Officer or Nursing Supervisor: He is a registered nurse who manage nursing staff, nursing procedures, and patient care at all integrated health facilities within the HZ.
- Pharmacist: The Pharmacist manages pharmaceutical services, including the procurement, storage, and distribution of medicines and medical supplies within the HZ.
- Laboratory Technician: Laboratory Technician oversee diagnostic and laboratory services, including the management of laboratory equipment and quality control procedures.
- Administrative Officer or Administrative Manager: He handle administrative tasks such as budget management, human resources, and general administration within the HZ.
- Data Manager: Data Managers are responsible for maintaining health records and data collection within the HZ.
- Community Health Officer and Health Promotion Officer: They often are representative of the Health Development Committee of the HZ. They work to engage with the local community and implement public health programs.
- Epidemiologist: In some HZs, an epidemiologist may be part of the team to monitor disease outbreaks, conduct surveillance, and support public health responses.

The specific roles and titles of HZMT members may vary, and some HZs may have additional personnel or specialized positions based on the unique needs and challenges of their area.

This team represents the foundation for the leadership that contributes to strengthening the health system at the operational level [30, 31].

The organization of DRC’s health system stems from the 1980s. It has been inspired by the Alma Ata (1978) recommendations regarding Primary Health Care’s organization, renewed through Astana Declaration (2018) [32, 33]. Some authors consider the design on which the Congolese health system is based to be one of the best thought-out and most robust in the context of sub-Saharan Africa: It has proven its resilience by surviving an environment of security, economic and governance crisis that has plagued the country throughout the 20 years following its independence in 1960 [34, 35]. Private faith-based healthcare organizations co-manage integrated health facilities with the Congolese State, filling a crucial role in healthcare delivery. Due to limited state funding, the health sector heavily relies on external Technical and Financial Partner (TFP) support, while households contribute significantly to operating funds and staff remuneration [36, 37].

### Selection of health zones

**Fig. 1:**
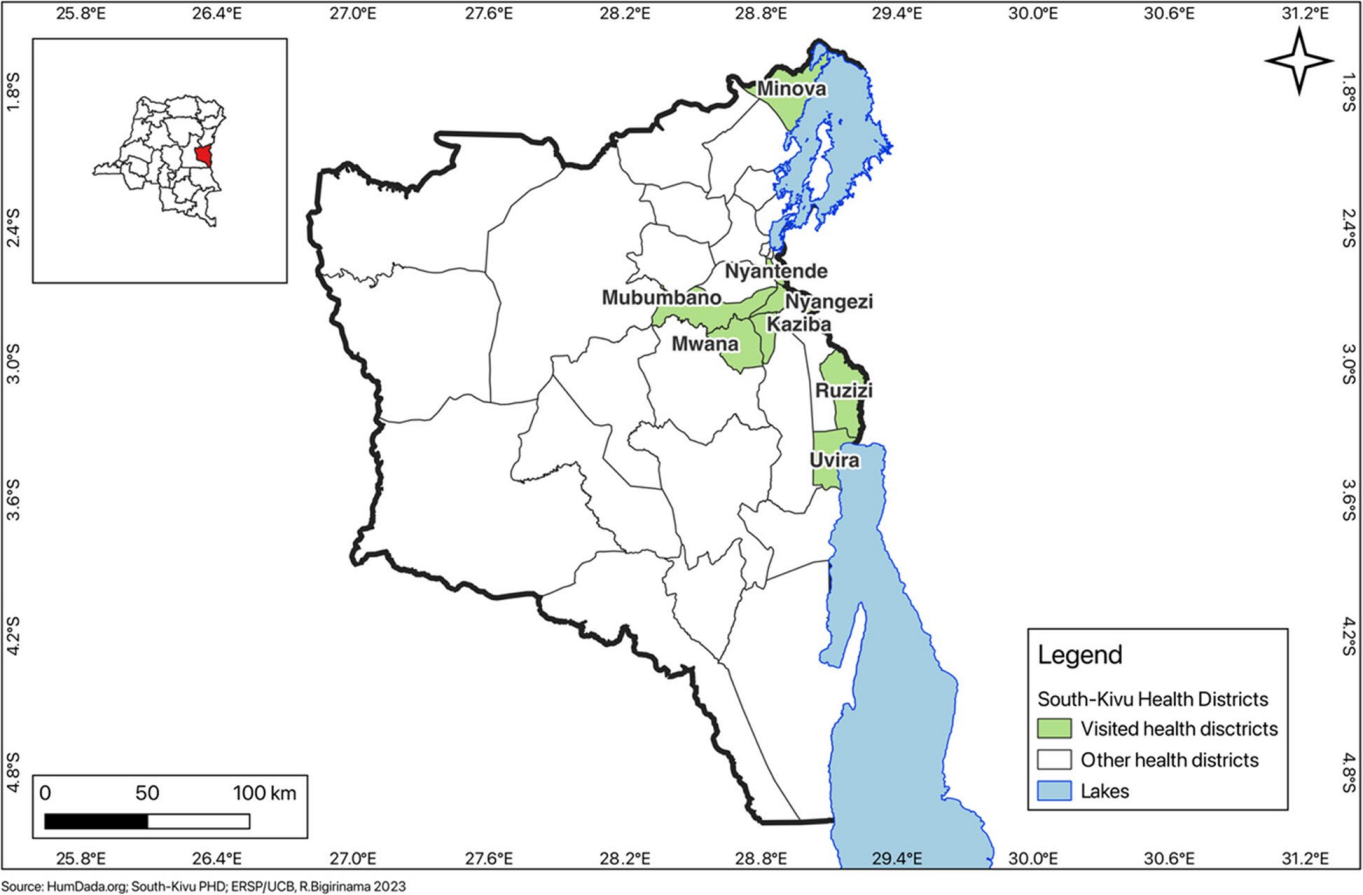
Study area

South-Kivu province is divided into a Provincial Health Division (PHD) with 34 health zones. Our study focuses on eight rural health zones, chosen based on geographical and security accessibility and encompassing 1,812,123 inhabitants (Table 1). We targeted zones receiving support from an international non-governmental organization’s health system strengthening program, ensuring a minimum level of functionality across all selected zones. International organizations play a pivotal role in sustaining the Congolese health system in this prolonged crisis setting, aligning their interventions with the country’s contextual priorities [30]. Our study’s HZs are spread over five of the eight territories that make up the province (Fig. 1): (i) Minova HZ, on the northern axis of the province (Kalehe Territory), (ii) three HZs (Kaziba, Mubumbano, Nyangezi) for the territory of Walungu located on the Central axis, (iii) Nyantede HZ for the territory of Kabare which forms a belt around the city of Bukavu, (iv) Mwana HZ for the territory of Mwenga located on the western axis of the province and (v) two HZs (Ruzizi and Uvira) for the territory of Uvira located on the southern axis.

**Table 1:** Health zones characteristics

Health Zones	Number of Heath Areas	Number of Health Centers	Number of Hospitals	Population in 2021	Administrative Status
Kaziba	16	16	2	144,471	Rural
Minova	21	21	6	379,352	Rural
Mubumbano	15	15	5	216,307	Rural
Mwana	15	15	3	154,455	Rural
Nyangezi	12	12	4	190,956	Rural
Nyantende	11	11	2	149,330	Urban-rural
Ruzizi	21	21	4	201,093	Rural
Uvira	22	22	10	376,159	Urban

### Study type

We performed a qualitative phenomenological study [38] to explore the opinions, perceptions, and attitudes of participants regarding leadership in their health zones. These participants were key leaders of the health zones, gathered in the Health Zone Management Team (HZMT): managers at the Health Zone General Referral Hospital and the Health Zone Central Office, and representatives of the local community involved in the Health Development Committee of the zone.

### Conceptual approach to leadership

Leadership is a concept that still varies greatly among experts. Thus, multiple theories have been described, drawing on one or other of its underlying dimensions [39]. For this study, we built our thematic framework inspired from the “Leadership Framework” conceptual approach to leadership of the United Kingdom’s National Health Service’s Leadership Academy [40, 41]. We developed six themes, four of which derive from the definition of the seven constitutive dimensions of the NHS’s Leadership Framework related to the managerial functions recognized in a functional HZMT (Theme 1 to Theme 4) (Table 2).

**Table 2:** Leadership thematic framework deducted from the NHS’s Leadership Framework

NHS’s Leadership Framework’s Leadership Dimensions	Study deducted themes
1) Demonstrating personal qualities	1) Perspective about Leadership
2) Working with others 3) Creating the vision	2) Internal collaboration within the HZMTs 3) Collaboration between the HZMTs and the Technical and Financial Partners (TFPs)
4) Managing services 5) Improving services 6) Setting direction 7) Delivering the strategy	4) Management and organization of the HZs
	5) Perception of the state of Leadership in the HZs 6) Leadership Strengthening Needs

### Data collection

In-depth interviews and non-participatory observations were used to collect data (Table 2). The data were collected by medical doctors who are senior researchers at the “Ecole Régionale de Santé Publique” school of public health of the Université Catholique de Bukavu, all of whom have extensive experience in qualitative data collection. As the study took place while the barrier measures against the Covid-19 pandemic were still in effect, these were respected throughout the data collection. Interviews were conducted in French and interviewers and participants were all fluent in that language. The data collection tools were pre-tested on a small scale on a population estimated similar to the one targeted by the study.

An interview guide exploring all six themes guided the individual discussions. Respondents matching the profiles were approached at their place of work, and the interview took place on the spot if the respondent gave consent and was available. Data collectors conducted an initial debriefing after each interview. The principle of saturation thus determined the number of interviews conducted in all eight HZs (Table 3).

Structured non-participatory observations of activities that could provide an appreciation of leadership practices and attitudes among managers were conducted whenever possible. Two grids lead the observations and were beforehand tested in similar contexts and populations.

The observations were of two types: observation of the managers in their daily work environment and observation of a routine work meeting of the members of the HZMT. The first one included observations of a clinical hospitalization ward round, a routine service delivery at the health center, a supervision session of a health center by members from the HZMT, or a normal day’s work at the health zone central office.

The observation themes in the grids were the technical settings of organizing the targeted activities, and the types of interactions during these activities. As attributes of the leadership dimensions “organization of work” and “communication” [41], these themes contributed to exploring two of the six themes of our thematic framework: Internal collaboration within the HZMT, and Management and organization of the HZ.

The targeted activities were observed whenever they coincided with the interviewers’ visit. The participants were not prepared beforehand about the observations. Their informed consent was obtained just before the onset of each observation.

The average interview time was 45 min. The average length of a routine meeting observation was 1.5 h, and that of observation of HZMT in their workplace was 8 h.

**Table 3:** Summary of types of information collected

Collection method	Profile						Total
Observations	Observation of a routine work meeting of the HZMT			Observation of managers in their work environment			
	05			06			11
Individual interviews	CMO	MD-GRH	AM-HZCO	HDCM	MSC	NS	
	03	07	02	09	01	03	25

*CMO* Chief Medical Officer of the HZ, *MD-GRH* Medical Director of the General Referral Hospital, *AM-HZCO* Administrative Manager of the HZ Central Office, *HDCM* Member of Health Development Committee of the HZ, *MSC* Medical Staff Chief of the GRH, *NS* Nursing Supervisor of the HZ

### Data analysis

Interviews were transcribed into Word documents. We performed a thematic analysis [42] with deductive and inductive approaches. The six themes initially established allowed for initial coding of the data. Then, within each theme, an inductive in vivo analysis identified various emerging sub-themes.

To describe leadership as perceived and experienced by the HZMTs, we applied triangulation of the information derived from purposive sampling for the interviews and for the observations, and for the selection of health zones. For the sake of anonymity, and to avoid any possibility of linking the identity of a respondent to a health zone, data were analyzed collectively for all eight health zones.

### Ethical considerations

The research protocol was submitted to and validated by the ethics committee of the Université Catholique de Bukavu under the reference UCB/CIES/NC/019/2021. Informed consent was obtained from each participant prior to any interview or observation. Data were collected and analyzed in complete confidentiality. All methods were carried out in accordance with relevant guidelines and regulations.

## Results

From the six initial themes, 13 sub-themes emerged (Table 4).

**Table 4:** Results overview

Main themes	Sub-themes
Perspectives about leadership	Governance
	Vision/positive influence
Internal Collaboration within the HZMTs	Positive collaboration
	Conflict and division
	Communication
Collaboration between the HZMTs and the TFPs	Communication and Planning
	Financial management
Management and Organization of the Health Zones	Health Zone management
	Organization of the zone
	Collaboration between HZs and PHD
Perception of the state of Leadership in the Health Zones	-
Leadership Strengthening Needs	Communication and planning
	Autonomy and decision-making power
	Capacity building

### Perspective about leadership

In analyzing this theme, two sub-themes emerged:

- Governance: Respondents consider a leader as someone capable of effectively organizing resources to meet the community’ needs. This leader must furthermore have a good grasp of public health standards and be able to hold his/her institution accountable to such standards.

*“A leader is a person who is trained in the health system and is able to organize or lead the health zone to find solutions to identified problems with the least amount of cost…” (P-4).*

*“…In the health system, (leadership) is how to organize things as it should be, the need to meet the expectations or needs of the community very well and needs of the health system.“ (P-10).*

*“…I think leadership is a team that mentors, that ensures that public health is really respected and effective at the health zone level…” (P-16).*

- Vision/positive influence: Respondents also mentioned that a leader with a clear vision is one who able to meet the expectations of the community. A leader inspires confidence in his collaborators and the local community. He /she can positively influence his/her team and steer them towards the realization of this common ideal of integral well-being.

*“Leadership, a leader is someone who leads a team, who leads a team for a common ideal or vision.“ (P-12).*

*“When we talk about leadership, we mainly see someone who has a vision and who has the ability to practically draw everyone towards him to bring them into that vision…” (P-18).*

### Internal collaboration within the HZMTs

There is some variability in internal collaboration among the members of the HZMTs, with notable differences between zones. Three sub-themes emerged from this theme:

- Positive collaboration: Some respondents appreciate the teamwork within their zones. The planning of activities is good, tasks are clearly distributed, and work is decentralized in term of execution. Financial management is transparent and honest, and personnel management is adequate.

*“…When we even do the planning of activities, we do it together to say that the health zone is functional…” (P-5).*

*“I appreciate the way they work internally, because normally we work in a decentralized way, each one does his job according to his attributions. I see that even in the absence of the zone’s chief medical officer, we organize the routine meetings of the management team; this already shows that there is delegation of power at the HZ level…” (P-9).*

- Conflict and division: Other respondents reported that there is conflict and division in their zones. Particularly when occasional activities generate dividends, some leaders step aside to share them without everyone’s participation. Some respondents report that these divisions have an impact on the governance of the zone, leading to irregularities in the implementation of HZ activities.

*“… I see that apparently Chief Medical Officers of health zones do not collaborate well with the subordinates. For the most part of the Chief Medical Officers of the zones that I have already met. Because they are the ones who make decisions themselves, especially regarding collaboration with partners. Sometimes they don’t give feedback on all the contacts they make with partners outside the team…” (P-15).*

*“… We do meetings together. We do all activities together. But when it comes to money, the management team is divided: “oh no, these people are from the hospital, and us we are from the zone…” and that sometimes causes problems. There are segregations when it comes to “war booty” …*” *(P-21).*

- Communication: Our observations of meetings in some areas indicate poor communication. In some HZs, observers noted meetings with an ambiance of a passive stillness where few people speak up, yet the resolutions made in the meetings were not contested. However, in other zones, communication in the meetings was smooth, with active participation of all, and collegiality in decision making.

### Collaboration between the HZMTs and the Technical and Financial Partners (TFPs)

By technical and financial partners (TFPs), we mean international non-governmental organizations and other partner institutions from multilateral collaboration engaged with the national health sector.

In this theme too, two different positions are noted among respondents, through the following two sub-themes:

- Communication and Planning: Some respondents are satisfied with the quality of communication between the TFPs and their zones. Communication is two-way and planning of activities is collaborative. Elsewhere, other respondents criticized the fact that the TFPs plan their activities without prior consultation with the HZMT, which is the reason for the failure of these activities given their non-contextualization to the needs of the zone.

*“The collaboration is good because in the team meetings we have reports (from partners) in relation to everything and (feedback) on their different advocacies.“ (P-12).*

*“As I told you we, i.e the management team, are in conflict with the partner (name of the partner) for not working well together because of selfish interests. People don’t collaborate; one hurts the other. Partnership is not respected.“ (P-4).*

- Financial management: Some respondents report a lack of transparency and traceability in the management of funds that partners grant to zones but still decide how these funds shall be used, sometimes resulting in situations of financial mismanagement for which the HZMT considers these partners directly responsible.

*“I think the problem between the HZMT and the technical and financial partners is a planning problem. We have our operational action plan in the zone, which is where the TFPs should join. But the TFPs plan activities outside this operational plan…” (P-5).*

*“…it’s true that there are always small concerns among certain financial partners who are not clear in their degree of collaboration with the zone, and therefore the transparency and traceability in relation to the funds or in relation to the support they bring to the health zone are not always well traced. They are not really very clear in the transparency, in the management…” (P-15).*

### Management and organization of the health zones

Three sub-themes emerged from this theme:

- Health Zone management: The management is not optimal. The issue of lack of internal cohesion within the teams and abuse of power by superiors is still noted in some HZ. This again takes the form of opaque management of funds, especially bonuses from extraordinary activities such as vaccination campaigns and large-scale campaigns of distribution of insecticide-treated bed nets. This mismanagement of the zone is also reflected in the administrative management of human resources, and delays in the payment of staffs’ salaries.

*“The management is not good, there is mismanagement of the HZ, the HZ Central Office is very badly managed. The chief himself manages the money, he himself is the cashier, the administrative manager, the accountant… So, everything is done in his office.“ (P-4).*

*“…there are several agents who do not receive (their remuneration), agents who are not matriculated, there are also many agents whose files are not complete, there are agents who do not have proper assignments and for whom regularization has not yet taken place…” (P-18).*

- Organization of the zone: A delay in carrying out the activities of the operational action plan of HZs is reported at all levels (health center, referral hospital or HZ central office) and is mainly the result of financial mismanagement or a failure to organize the work plan. This disorganization of the zone is also found in the organization of different healthcare packages at the first and second lines of care: in some HZs, there is no longer a clear demarcation between structures supposed to offer first- and second-line care packages; some front-line facilities anarchically offer services that are exclusive to second-line facilities. The lack of communication is also translating into tensions between facilities’ co-managers over staff assignment and other managerial decisions.

*“…In relation to the management of activities, I can say that there are times when the supervision schedule is not followed, for example, at the referral hospital in (name of the zone) and at the level of the primary structures, the health centers. Therefore, there is a lack of respect of the supervision schedule that was established for the activities” (P-3).*

*“… A health center that doesn’t respect its minimum package of activities; it does what it wants. Imagine a health center that performs blood transfusions…” (P-18).*

*“Here we have a general referral hospital which has a private manager that always poses the problem of co-management with the Ministry of Health… They can bring in someone who he is a teacher or an agronomist, they appoint him as the hospital’s administrative manager. There is really no collaboration in the assignment of officers who manage the hospital…” (P-5).*

- Collaboration between the zones and the Provincial Health Division (PHD): Some respondents reported close collaboration between the zones and the PHD. However, others found that the management of this collaboration was not optimal. In particular, they criticized the abuse of power by the PHD in the form of interference in the management of HZ staff and the planning of HZ activities. In addition, the zone’s expectations in terms of capacity-building support from the PHD are not always met.

*“…PHD doesn’t support the HZs like they used to when we had a lot of supervisions and that helped us. But now they’re once or twice a year and if we got those supervisions once a quarter it would be fine…” (P-2).*

*“…there are still some irregularities when it comes to some activities that are practically the responsibility of the HZ Chief Medical Officer or the management team but are carried out by PHD managers… Examples of the opening of private health centers that do not meet any standards…” (P-18).*

### Perception of the state of leadership in the health zones

The perception of the leadership of the HZMT varies among respondents. Some see effective leadership, with initiatives to bring together different local leaders, while others point to difficulties in managing unmotivated and resistant staff. In addition, issues of lack of cohesion within the HZMT, and the vertical approach of Technical and Financial Partners in their support to zones, were raised as factors that weaken the quality of leadership within the HZMT.

*“In the public health activities, leadership is conducted: the HZMT plans a meeting where it will call on all local community leaders, as well as influential leaders, to take certain (measures) together in the (management of) certain problems in society…” (P-1).*

*“It is not easy for (the zone leaders) to impose themselves on unmotivated staff. Often there is resistance, and they often have difficulty managing staff. This problem has a negative impact on the implementation of activities, but they do their best to keep us within the standards.” (P-6).*

*“… And so instead of the different partners aligning themselves with the planning of the health zone, we feel at times that it’s the health zone that practically aligns itself with partners objectives and planning. And so, that’s kind of the big constraint in practice.” ( P-18).*

### Leadership strengthening needs

This theme explored respondents’ views on the need for leadership development in the area. The following three sub-themes emerged:

- Communication and planning: Respondents emphasized improving communication and collaboration between the members of the HZMT, the PHD and technical and financial partners. This would restore cohesion and promote planning adapted to the realities of the zone.

*“I would recommend that when there is an activity, that the team organizing at the provincial level take into account the planning at the grassroots level.“ (P-5).*

- Autonomy and decision-making power: Respondents expressed the need for more autonomy in management and decision-making at the health zone level.

*“The first recommendation is to give more responsibility to the HZ Medical Officers. They know what they have to do, but at times the PHD want to treat them like little children and that’s something that doesn’t go down well…” (P-18).*

- Capacity building: Respondents expressed a need to strengthen the clinical and managerial capacities of the various local actors to better manage the health zone, improve its performance and the health status of the local community. Some respondents emphasized that better work planning and organization skills would contribute to the achievement of the health zones’ operational objectives. They felt that having a daily work plan to help manage time on the job would help them perform better. In addition, observations of the managers in their daily work environment reported an overall respect for the start and end times of work. However, of the average 8 h spent at work, less than half (an average of 3 h) is spent actually working, with time at work coexisting with untimely comings and goings, unscheduled private visits, and frequent and sometimes long breaks outside of work on the phone or in conversation with colleagues on site. Unlike meetings where the agenda is set in advance, in none of the observations made was there any prior preparation of the daily work plan, nor were there any specific time slots set aside for various tasks.

*“You know, you can’t punish someone for what they don’t know, I can say that trainings are always important because if you are trained in leadership and human resource management it can always build capacity.“ (P-9).*

*“Yes, it’s the training. Especially training in primary health care management could help the zone to know how to apply or revitalize and sustain leadership in the health zone, but also the collaboration of partners.“ (P-20).*

## Discussion

This study aims to contribute to strengthening the DRC’s health system by addressing the leadership at the operational level. Based on the NHS leadership framework [41], we have explored the question by examining key functions that are closely tied to the mission expected from health zone responsible as part of their HZMT roles.

### A rather transactional perspective of leadership

The findings suggest that HZ managers are inclined to consider leadership from a transactional standpoint. Whereas transformational leadership implies a high level of coordination, communication, and cooperation [43–46], transactional leadership relies primarily on reward and sanction as motivational tools. Many participants equated leadership with strict management and compliance with established procedures. However, this stands in stark contrast to transformational leadership, which some authors argue could be better suited to such settings. Indeed, looking at leadership perceptions in sub-Saharan Africa, many researchers have concluded that this latter type provides a model particularly relevant in unstable and crisis-ridden environments [43, 44, 47–49].

Leadership concept is dynamic, varying according to context and individual perspective. It is widely associated with effective management, procedural rigor, knowledge, initiative-taking and even charisma [39, 50]. Keywords defining leadership identified in published literature and quoted by most of the respondents indicate, at the very least, that they have been exposed to or updated on the subject. As such, their understanding may reflect a broader influence from administrative authorities and bureaucracy. Such influences might derive from the higher hierarchical levels of the healthcare system, at which compliance with prescribed procedures is generally emphasized or imposed [51].

### Leadership and organizational culture

While the DRC’s healthcare system was devised to enable autonomous operation and optimal provision of primary healthcare by the health zones [30], the latter suffer from financial difficulties [52], which emerged regularly in our respondents’ answers as sources of contention. Any opportunity to supplement salaries through extraordinary activities turns into a potential windfall for constantly underpaid health workers. As a result, rifts within these already fractured communities widen, hindering team cohesion behind a common vision as well as transposing leadership principles into practice [13, 43]. Similar frustrations were voiced by several of the interviewees. Moreover, the literature abounds in showing just how detrimental such interpersonal tensions among healthcare personnel are to both patient care and health institution management [53, 54]. Leadership requires dialogue, transparency, and a collective project to foster an organizational culture of good governance and to counterbalance power dynamics. Collectively, these elements contribute to improving health system performance and achieving health system objectives [1, 55].

Observations revealed inadequate time management. Untimely visits, inappropriate cell phone use, and a lack of a precise daily work plan can harm performance by increasing cognitive load and distracting employees [56, 57]. As new communication technologies integrate into daily work, experts recommend regulating social media use rather than banning it [57, 58].

### Working with Technical and Financial Partners (TFPs)

Our interviews revealed interference between HZs operational planification and the interventions of the TFPs. The Achilles’ heel of developing countries’ healthcare systems has been frequently considered to be their dependence on external aid, which finances the bulk of health promotion activities [59–61]. While international partner interventions are essential and invaluable for low-income country health programs, their prescriptive nature may impede empowerment and leadership development efforts in such contexts [60, 61].

The respondents spoke of the damaging influence of provincial interference in reducing their decision-making autonomy as well as their motivation, particularly in regulating the operation of health structures and human resources management. The DRC healthcare system is organized in such a way as to offer managers at operational level a comfortable decision space. While theoretically operational management is incumbent on HZs [30, 62, 63], several authors have pointed out the opposite in practice [64, 65]. This underscores the clear imperative to bolster both operational and national leadership.

### Strengthening leadership

We analyzed respondents’ views on leadership development needs across the seven domains of the NHS’s Leadership Framework [41]. In the three sub-themes identified from the results, respondents expressed the need for enhancing personal qualities (knowledge, skills, and attitudes), teamwork, service management, direction setting, and strategy delivery.

While the DRC’s Ministry of Public Health, in collaboration with certain Technical and Financial Partners (TFPs), regularly provides capacity-building training for healthcare system personnel, these trainings typically consist of ad hoc, unstructured briefings that may not systematically reach all levels of the healthcare system [27]. Further research is required to assess the actual impact of these trainings on enhancing the performance and functionality of health zones. Prior studies on leadership development programs in sub-Saharan Africa have highlighted diverse leadership development needs, encompassing conceptual leadership, capacity building, theoretical knowledge, health policy, service management, emergency planning, communication, advocacy, and experiential learning through internships with local mentors or Northern institutions [26, 66, 67].

### Study limits

Some limitations should however be considered. Firstly, the observation reported by this study concerns the year 2021: in the context of the DRC, the study may not fully capture the dynamic challenges faced by health managers in such an unstable context. It should also be noted that individual interviews can introduce biases due to the subjective experiences of the participants, and observations can be biased by the observer effect (Hawthorne effect) [68]. We hope that the combination of collection methods will have minimized this aspect to some extent, as will the analysis of the data by a large team of researchers.

## Conclusion

The mission of HZMTs in DRC is to provide effective leadership to manage and organize the health zone, foster collaboration, increase decision-making autonomy and continuously develop leadership. The HZMT plays a central role in delivering health care, managing resources, and optimizing operations to serve the community. However, our study highlights the challenges of operational leadership in DRC’s protracted crisis areas.

Financial constraints, interference in planning and limited decision-making autonomy are obstacles to leadership. We emphasize that DRC’s health zones already have the necessary structure for the operational level of the health system, and the HZMT has tools and standards to fulfill its mission. But they are not fully exploited, and the potential of this robust system is under-utilized. We believe that addressing the identified weaknesses could lead to significant improvements in the health zone’s results.

## Data Availability

Data generated and analyzed during the current study are not publicly available due to confidentiality restrictions, but anonymized transcripts and observation grids are available from the corresponding author upon reasonable request.

## Abbreviations

DRC: Democratic Republic of Congo
HZ: Health Zone
HZMT: Health Zone Management Team
NHS: National Health Service (of the United Kingdom)
PHD: Provincial Health Division
TFP: Technical and Financial Partners
WHO: World Health Organization
